# Efficacy of 1064‐nm Picosecond Laser in the Treatment of Lichen Planus Pigmentosus: A Split‐Face Randomized Controlled Trial

**DOI:** 10.1111/jocd.70846

**Published:** 2026-04-07

**Authors:** Suthinee Rutnin, Supachak Smitthisakda, Namthong Wittayabusarakam, Tanat Yongpisarn, Nawara Sakpuwadol, Kunlawat Thadanipon

**Affiliations:** ^1^ Division of Dermatology, Faculty of Medicine Ramathibodi Hospital Mahidol University Bangkok Thailand; ^2^ Department of Clinical Epidemiology and Biostatistics, Faculty of Medicine Ramathibodi Hospital Mahidol University Bangkok Thailand

**Keywords:** 1064‐nm picosecond laser, lichen planus pigmentosus (LPP), pigmentary disorders, split‐face trial

## Abstract

**Background:**

Lichen planus pigmentosus (LPP) is an uncommon variant of lichen planus that causes visible pigmentation and significantly impacts quality of life. Current treatments for LPP are limited, with inconsistent outcomes from topical, oral, and laser therapies. The 1064‐nm picosecond laser has shown potential in managing pigmented skin disorders; however, evidence regarding its effect on LPP remains scarce.

**Objectives:**

This study aimed to evaluate the efficacy, safety, and patient satisfaction of the 1064‐nm picosecond laser in treating LPP.

**Methods:**

The study was a randomized, split‐face controlled trial. Twelve patients with biopsy‐confirmed LPP were enrolled, with one side of each participant treated with four sessions of the 1064‐nm picosecond laser at monthly intervals. The Modified Dermal Pigmentation Area and Severity Index (mDPASI) and melanin index (MI), assessed by three‐dimensional imaging, were measured before each treatment and at 1, 3, and 6 months following the last treatment. Physician global assessment (PGA) and patient satisfaction scores were assessed at 1 and 6 months following the last treatment. Additionally, adverse events were recorded.

**Results:**

A total of 12 patients with LPP, aged 55 ± 11.1 years, were recruited, and 11 completed the study. During the 6 months following the end of laser treatment, no significant differences were found in MI, mDPASI, or PGA between treatment and control groups. However, satisfaction scores showed a significant improvement on the treated side compared to the control. The mean overall pain score was 3.70 ± 1.72, with no major adverse events reported.

**Conclusion:**

The 1064‐nm picosecond laser demonstrated limited effectiveness in improving LPP clinical outcomes. Further studies with sham‐controlled trials and larger sample sizes are needed to optimize treatment parameters and sessions, and to validate the role of the picosecond laser as a therapeutic option for LPP.

## 1 Introduction

Lichen Planus Pigmentosus (LPP) is an uncommon variant of lichen planus which is frequently seen in individuals with darker skin types (Fitzpatrick skin types III‐V), and is more commonly seen in females. Clinically, it presents as slate‐gray macules and patches, typically found on the face, neck, and flexural areas [1, 2]. Histologically, LPP is characterized by lichenoid dermatitis, epidermal hyperkeratosis, hypergranulosis, and moderate to severe pigmentary incontinence [3]. Patients with LPP experience substantial psychosocial impact due to cosmetic disfiguration and have a significantly impaired quality of life [4, 5].

Currently, there is no standard treatment for LPP [6], and existing studies are small and non‐randomized. Tacrolimus 0.03% ointment led to appreciable lightening in 53.8% of 13 patients in one study [7], while low‐dose isotretinoin (20 mg/day) led to mostly moderate (55.7%) and good (21.8%) improvement in 27 patients [8]. Using toning Q‐switched Nd:YAG laser (1064 nm, fluence 3 J/cm [2], spot size 6 mm) on single 5 × 5 cm [2] lesions biweekly for 6 sessions achieved mean improvement of 25.7% in nine patients [9]. Another study using the Q‐switched Nd:YAG laser at 4–8 week intervals for 5–6 sessions on facial lesions yielded > 90% improvement in 38.5% of 13 patients [10]. These findings suggest that laser therapy may offer some benefit in LPP, but the variability in outcomes highlights the need for further investigation into alternative laser approaches.

Picosecond lasers are a novel technology that deliver shorter pulse durations in the picosecond range compared with Q‐switched laser devices, which employ nanosecond pulses. This allows faster energy delivery, resulting in higher target pressure with less thermal diffusion [11, 12], and induces photomechanical effects that break down pigment particles [13]. Picosecond lasers have been shown to be efficacious in targeting dermal melanophages in melasma [14], and there has been a case report of significant improvement in LPP following picosecond laser treatment [15]. We aimed to investigate the efficacy and safety of picosecond laser treatment in patients with LPP.

## 2 Material and Methods

### 2.1 Study Design

This prospective, split‐face, randomized, controlled, evaluator‐blind study was conducted at a university‐based hospital from November 2022 to February 2025. The protocol conformed to the guidelines of the Declaration of Helsinki. Written informed consent was obtained from all participants prior to enrollment.

### 2.2 Participants

This study included patients aged more than 18 years old with biopsy‐proven LPP with bilateral, symmetric lesions on the face and/or neck. Eligible participants were required to have stable disease (the absence of new lesions or darkening of existing lesions, regardless of whether the patient was currently using topical and/or oral medications) for at least 6 months before enrollment. Prospective participants were excluded from the study if they had (1) co‐occurring skin infection, active skin inflammation, acne vulgaris or open wounds at the treatment site, (2) a history of immunodeficiency, skin cancer, coagulopathy, or recurrent herpes infection, (3) pregnancy or breastfeeding at the time of enrollment, and (4) a history of laser treatment within 6 months before the study.

### 2.3 Treatment

A computer‐generated, block randomization of the facial side to receive 1064‐nm picosecond laser treatment was concealed in sequentially numbered, opaque, sealed envelopes. According to the block randomization, one side of each participant's face was assigned to receive treatment, while the untreated side served as the control group.

The treated side underwent four sessions of treatment with a 1064‐nm picosecond Nd:YAG laser (Enlighten System; Cutera Inc.) at 4‐week intervals using the following parameters: spot size 10 mm, fluence 0.4–0.6 J/cm [2], frequency 10 Hz, and two to three passes. The clinical endpoint of laser treatment was mild erythema. The untreated side served as the control. The participants were advised to avoid sun exposure and to continue their previous systemic and/or topical treatments equally on both the treated and control sides to minimize the risk of worsening on the control side during the study period.

**FIGURE 1:**
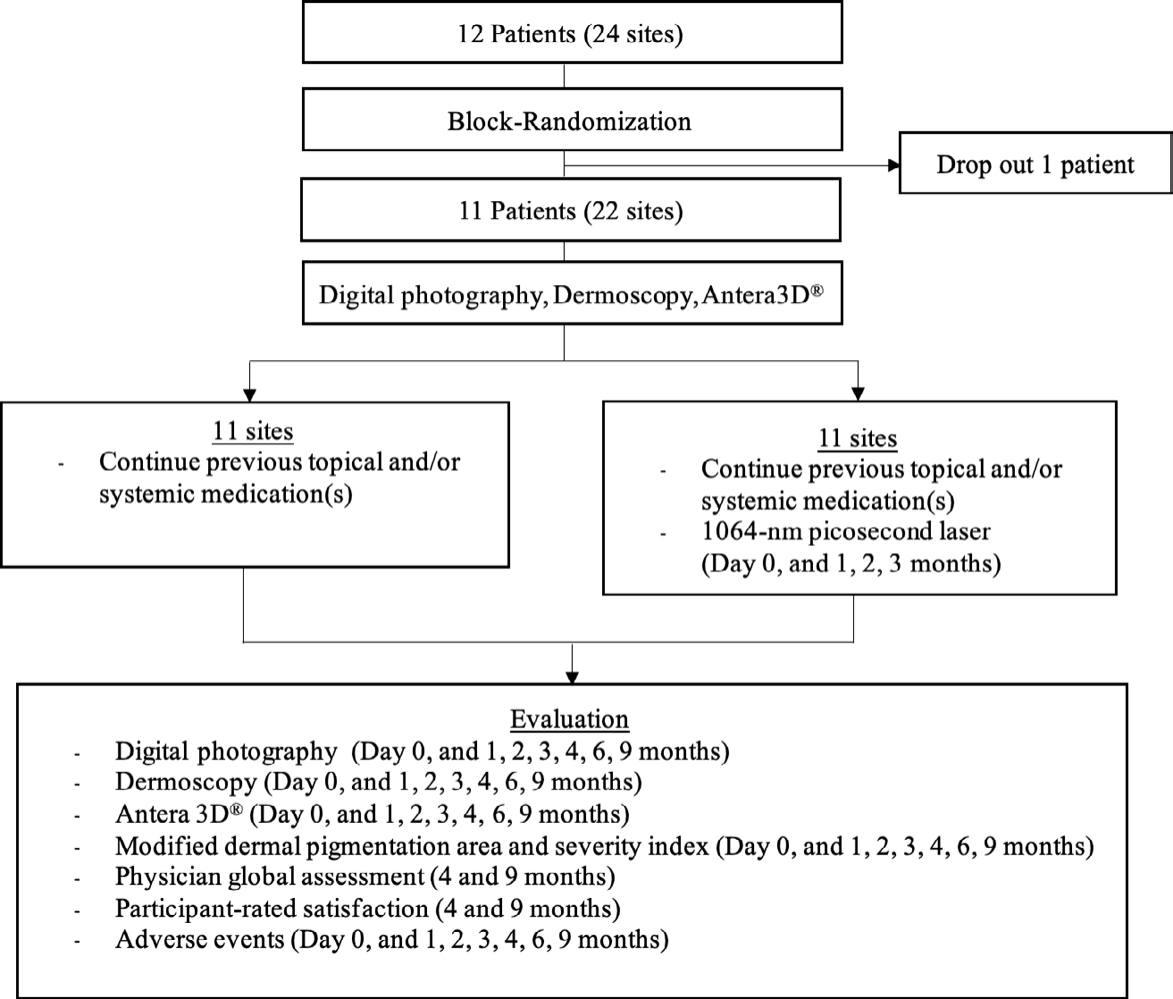
Protocol flowchart.

To minimize pain during the laser procedure, continuous air cooling (Cryo 6; Zimmer Aesthetics Ltd.) was applied throughout the session. After each treatment, a cold compress was applied for 10–15 min to reduce discomfort. Participants were instructed to apply broad‐spectrum sunscreen (SPF 50+, PA+++) once daily in the morning and after each treatment session. The researchers provided additional laser treatment on the untreated side in cases where patients developed visible asymmetry in pigmentation between the treated and untreated sides after the study until lesions on both sides were balanced. The study flow diagram is shown in Figure 1.

### 2.4 Outcome Evaluation

**FIGURE 2:**
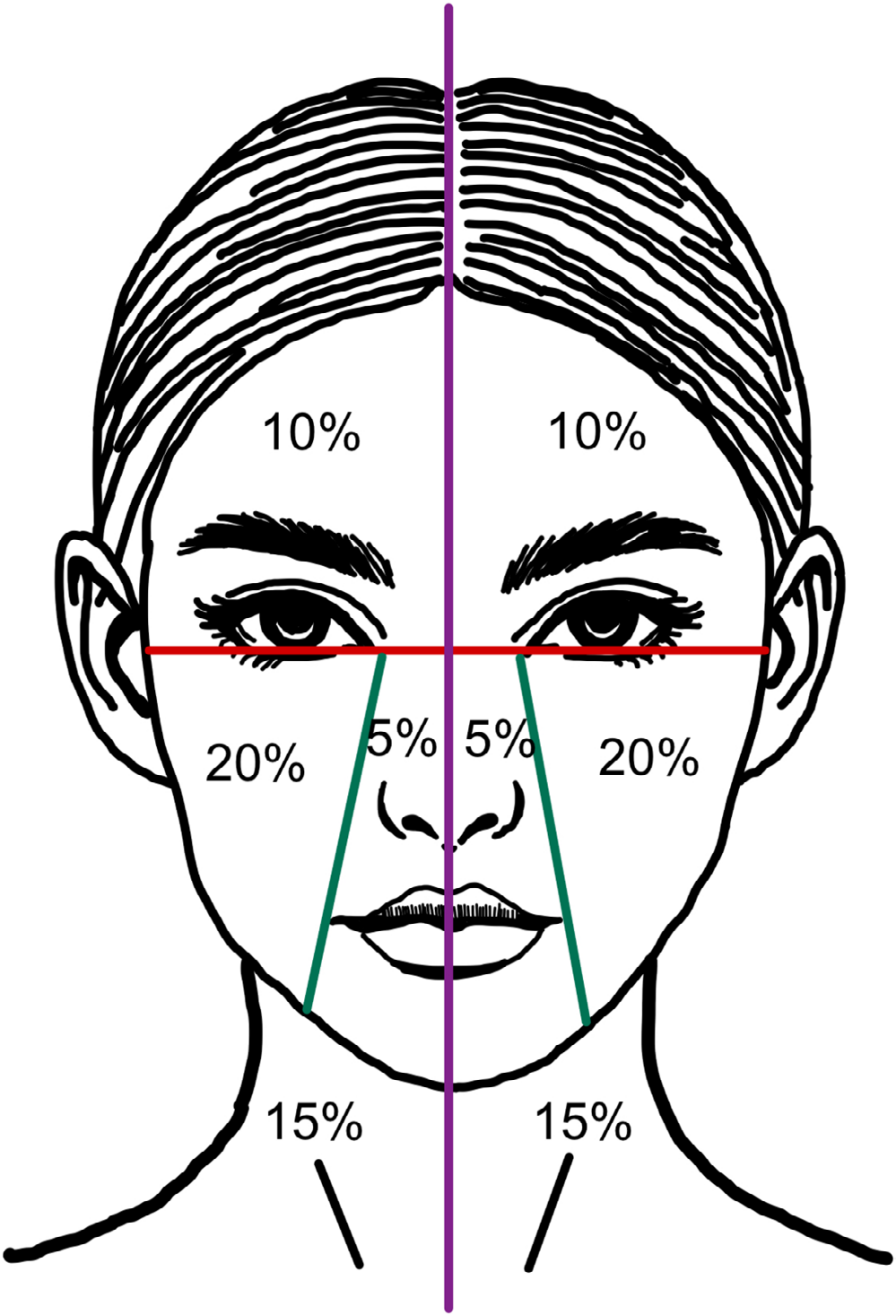
Facial and neck area division based on our modified dermal pigmentation area and severity index (mDPASI). The face is divided into two halves, with each half segmented into four regions: The forehead (10%), cheek (20%), central face (nose and perioral area, 5%), and neck (15%). The mDPASI score is calculated as follows: 1× (percentage of forehead involvement × grade) + 2× (percentage of cheek involvement × grade) + 0.5× (percentage of central face involvement × grade) + 1.5× (percentage of neck involvement × grade).

The primary outcome was the improvement in pigmentation of lichen planus pigmentosus (LPP), assessed using the Modified Dermal Pigmentation Area and Severity Index (mDPASI), the melanin index (MI), and the Physician Global Assessment (PGA). The mDPASI is a modified version of the Dermal Pigmentation Area and Severity Index (DPASI) [16, 17]. In this study, the mDPASI was developed by reducing the weighting factors applied to different facial regions, with most areas adjusted to approximately 50% of the original values. Specifically, the forehead weighting was reduced from 2 × to 1 ×, and the central face from 1× to 0.5×, whereas the cheek and neck regions remained unchanged. The intensity of pigmentation was classified into five grades: Grade 0, no change in color or normal dermoscopic pattern; Grade 1, light brown discoloration and/or a dotted pattern on dermoscopy; Grade 2, bluish or violaceous pigmentation and/or a Chinese letter or semi‐arcuate pattern; Grade 3, slate‐gray to brown pigmentation and/or a reticulate pattern; and Grade 4, dark brown to black discoloration and/or a diffuse pattern on dermoscopy. The mDPASI was calculated by multiplying the percentage of the affected area in each region by its corresponding weighting factor and the grade of pigmentation severity. The modification aimed to better assess the pigmentation severity in a split‐face study (Figure 2). A board‐certified dermatologist, blind to treatment allocation, assessed each patient using this index to compare the treated and untreated sides of the face. The melanin index was measured using a three‐dimensional skin imaging device (Antera 3D; Miravex Limited). Both the mDPASI and melanin index were evaluated at baseline, each laser treatment visit, and at 1, 3, and 6 months post‐treatment. The PGA is a five‐point scale evaluating overall improvement in pigmentation. It was assessed by a blind board‐certified dermatologist through photographic comparison at 1 and 6 months post‐treatment, with the following scores: 0, worse than before treatment; 1, < 25% improvement; 2, 25%–49% improvement; 3, 50%–74% improvement; 4, 75%–100% improvement.

Secondary outcomes included participant‐rated satisfaction using a visual analog scale (VAS) (range 0–10), assessed at 1 month and 6 months post‐treatment, and the incidence of any adverse events, such as burning or pruritus, evaluated at each visit.

### 2.5 Statistical Analyses

Categorical data were described as numbers and percentages, and continuous data as the mean and standard deviation (SD) or median and interquartile range (IQR). mDPASI and melanin index were compared between the laser‐treated and untreated sides using mixed‐effects linear regression. PGA was analyzed using the marginal homogeneity test. Additionally, participant‐rated satisfaction scores were compared between treated and untreated sides using a paired *t*‐test. Two‐sided *p*‐values < 0.05 were considered statistically significant. All statistical analyses were performed using Stata/SE version 18 (StataCorp LLC).

## 3 Results

### 3.1 Characteristics of Participants

A total of 12 participants were recruited, with 11 completing the study, resulting in 22 facial sites. One participant withdrew from the study due to inconvenience in attending follow‐up. The mean ± SD age of participants was 55 ± 11.2 years, with a majority being female (81.8%).

Regarding Fitzpatrick skin type, most participants had either type IV or type V in equal proportions (45.5% each), while only one participant (9.1%) had type III. The median (IQR) disease duration was 24 (24) months. The most commonly affected areas were the cheeks (90.9%) and neck (90.9%), followed by forehead (63.6%) and central face (27.3%).

### 3.2 Previous Treatments

Prior to recruitment, four participants (36.4%) had received only topical treatment, while seven participants (63.6%) had received both topical and systemic treatments. The specific topical treatments varied among individuals, with participants receiving one or a combination of the following: hydroquinone, calcineurin inhibitors, whitening agents, steroids, retinoids, alpha hydroxy acids, tranexamic acid, and azelaic acid. For systemic treatment, each participant had received either oral acitretin or oral tranexamic acid. Table 1 presents the characteristics of participants.

**TABLE 1:** Demographic characteristics of participants.

Characteristics	Value
Age, mean ± SD, year	55 ± 11.2
Gender, *n* (%)	
Male	2 (18.2)
Female	9 (81.8)
Onset of disease, median (IQR), month	24 (24)
Fitzpatrick skin type	
III, *n* (%)	1 (9.0)
IV, *n* (%)	5 (45.5)
V, *n* (%)	5 (45.5)
Location of the disease	
Forehead, *n* (%)	7 (63.6)
Cheek, *n* (%)	10 (90.9)
Central face, *n* (%)	3 (27.3)
Neck, *n* (%)	10 (90.9)
Previous treatment, *n* (%)	
Combination treatment	7 (63.6)
Topical treatment	4 (36.4)
Baseline mDPASI, mean ± SD	
Treatment side	7.16 ± 4.04
Control side	7.19 ± 4.01

Abbreviations: IQR, interquartile range; mDPSAI, modified Dermal Pigmentation Area and Severity Index; mVSS, modified Vancouver scar scale; n, number; SD, standard deviation.

### 3.3 Assessment of Hyperpigmentation (mDPASI, Melanin Index and PGA)

**FIGURE 3:**
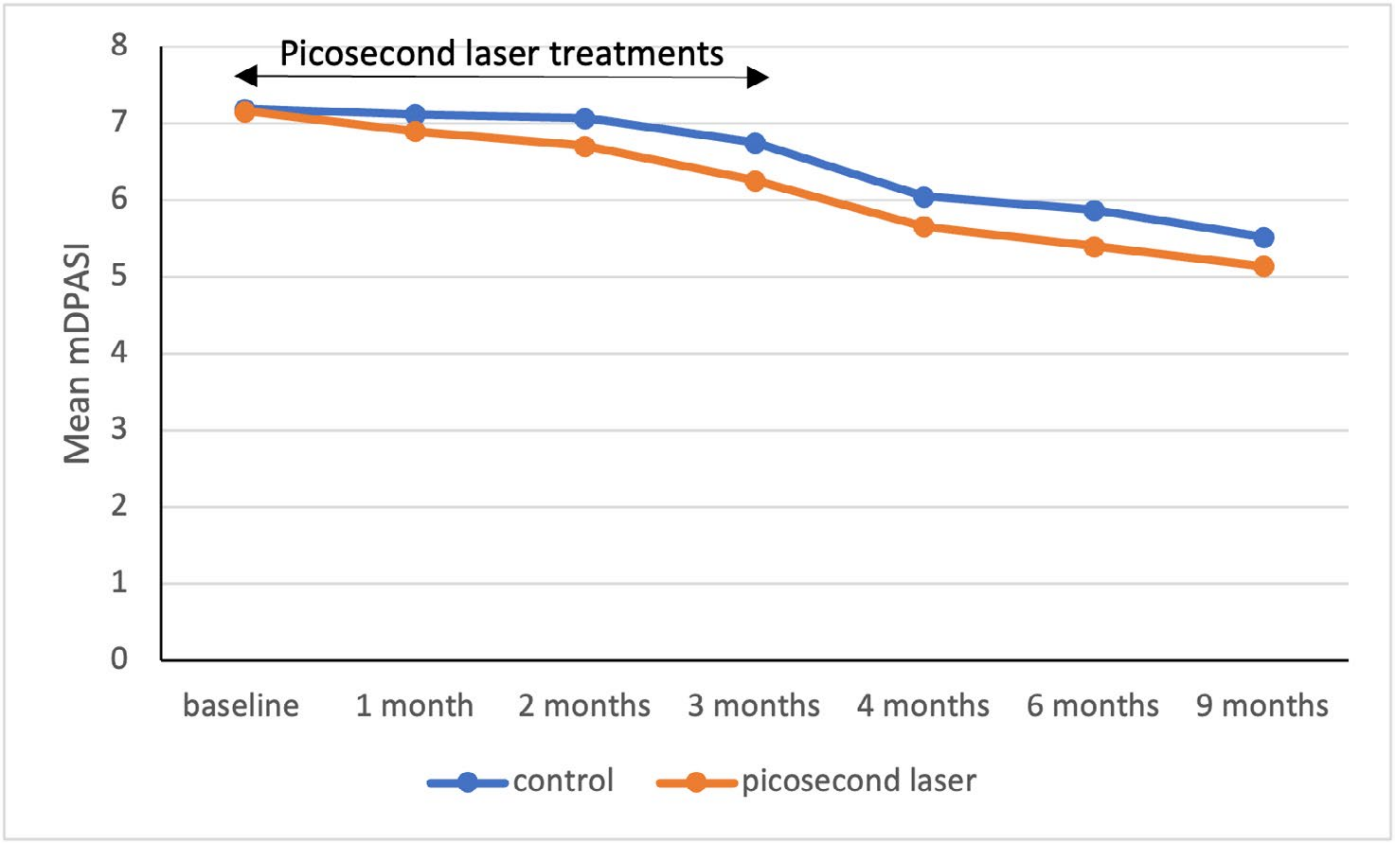
Graph comparing the mean modified dermal pigmentation area and severity index between picosecond laser‐treated sides and control sides at each visit.

During the study period, the 1064‐nm Nd:YAG picosecond laser‐treated side showed no statistically significant reduction in mDPASI scores compared to the control side at any time point (*p* > 0.05) (Figure 3) (Table S1).

Regarding the melanin index, a statistically significant difference was observed at 2 months on the forehead (*p* < 0.05) (Table S2); thereafter, pigmentary differences at each disease location were not statistically significant between the treatment and control sides, consistent with both mDPASI and PGA scores (Tables S3 and S4).

PGA scores were assessed at 1 and 6 months post‐treatment. Although there were no statistically significant differences between the treatment and control sides (*p* > 0.05), the laser‐treated side demonstrated higher mean PGA scores compared to the control side at both 1 month (1.3 vs. 1.1) and 6 months post‐treatment (1.9 vs. 1.7), with four participants (36%) achieving ≥ 50% improvement in pigmentation.

**FIGURE 4:**
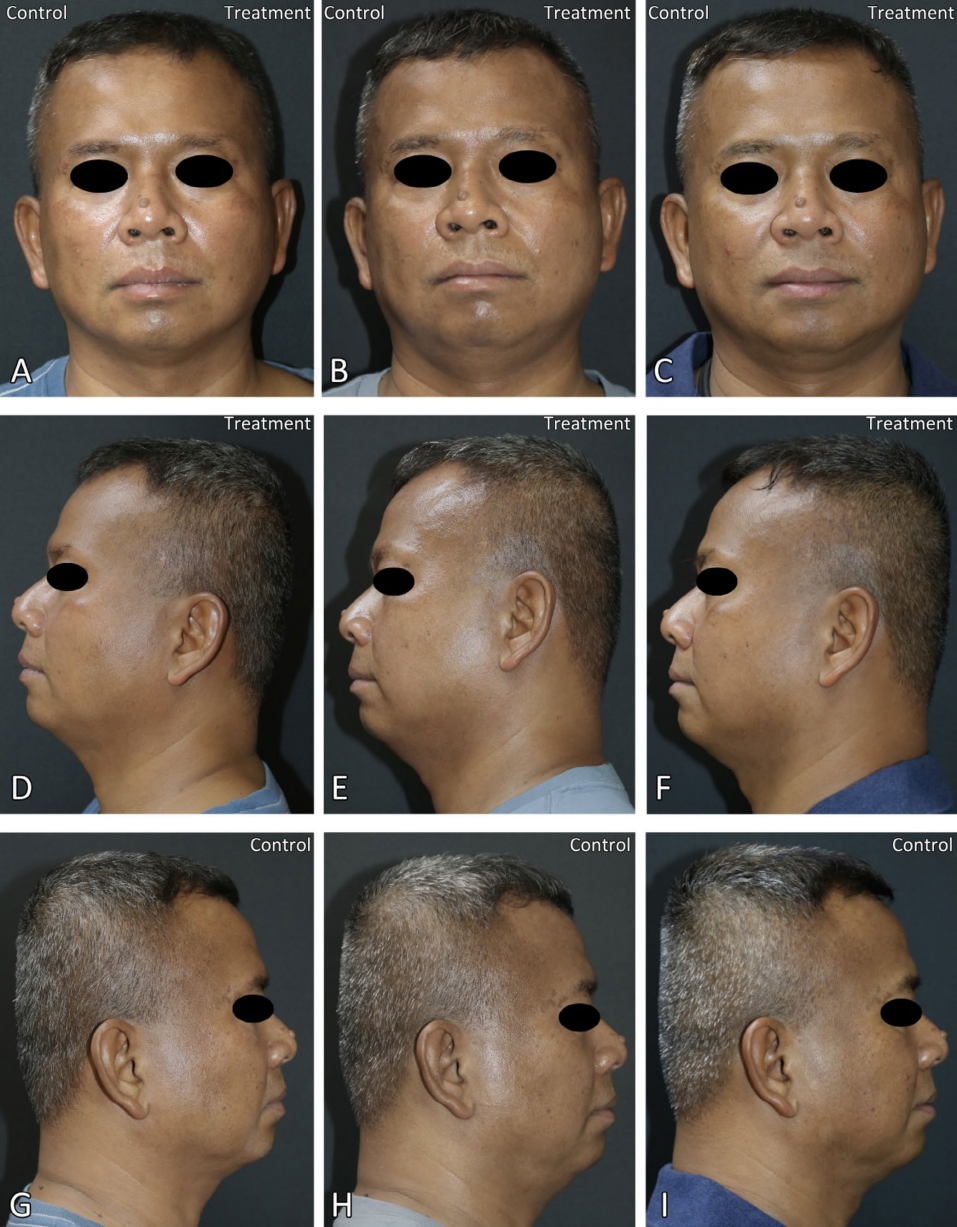
Digital photographs of participant number 3 at baseline (A, D, G), 1 month (B, E, H), and 6 months post‐treatment (C, F, I). Treatment and control sides are labeled accordingly in each image.

**FIGURE 5:**
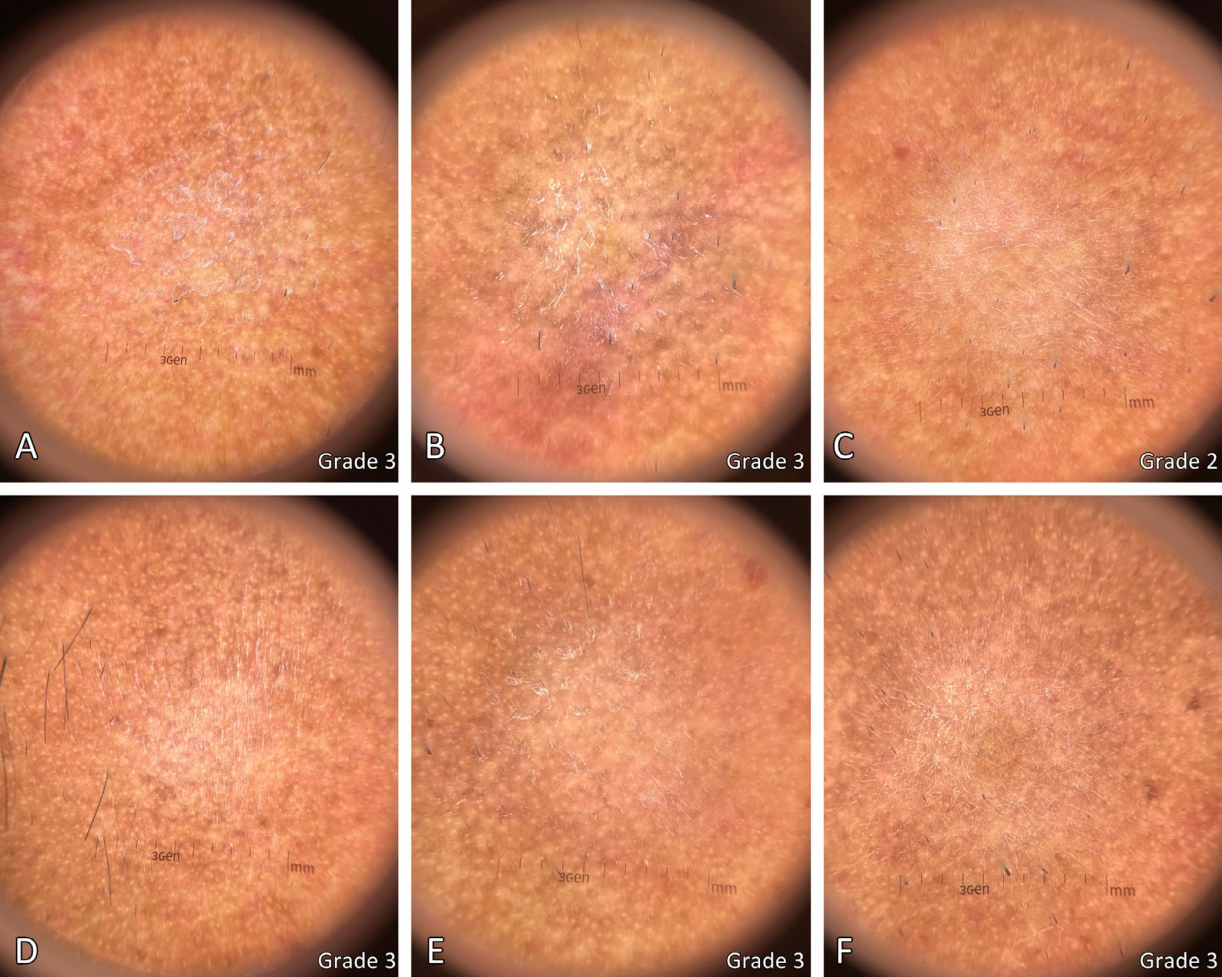
Dermoscopy images of the patient from Figure 4, comparing the 1064‐nm picosecond laser‐treated side at baseline (A), 1 month (B), and 6 months post‐treatment (C), and the control side at baseline (D), 1 month (E), and 6 months post‐treatment (F). Pigmentation severity grading is shown in the bottom right corner of each image.

Figures 4 and 5 show clinical and dermoscopic images from participant number 3, a 49‐year‐old man with symmetrical slate‐gray to brown macules and patches on the face for 3 years. Clinical improvement on the laser‐treated side compared to the control side was observed in the clinical photographs 1 month and 6 months post‐treatment (Figure 4), corresponding to pigmentary reduction in the dermoscopic images (Figure 5).

### 3.4 Participant‐Rated Satisfaction and Adverse Events

At 1 and 6 months post‐treatment, participant‐rated satisfaction (VAS, 0–10) was significantly higher on the laser‐treated side compared to the control side at both 1 month (6.9 vs. 5.2, *p* = 0.005) and 6 months post‐treatment (7.7 vs. 5.8, *p* = 0.008).

No major adverse events were reported. Seven participants (63.6%) experienced a burning sensation and three patients (27.3%) reported pruritus. Both adverse events resolved within a few hours. The mean ± SD pain score among participants was 3.7 ± 1.7.

## 4 Discussion

In the present study, we conducted an evaluator‐blind, split‐face, randomized controlled trial to evaluate the efficacy, safety, and patient satisfaction of a 1064‐nm picosecond Nd:YAG laser using a low‐fluence toning protocol for the treatment of LPP. Our findings demonstrated no statistically significant improvement in pigmentation at the end of the study, as assessed by mDPASI, melanin index, and PGA scores, compared to the control side; however, an early significant reduction in melanin index was observed at 2 months on the forehead.

Picosecond lasers are characterized by a shorter pulse duration and reduced thermal effect compared to Q‐switched lasers [11], allowing fragmentation of pigmented structures with minimal collateral damage [12, 18, 19]. They are considered the gold standard for tattoo removal and have also been explored in various pigmentary disorders such as nevus of Ota [20], Hori's macules [21], and melasma [14]. In LPP, chronic interface dermatitis leads to damage to basal keratinocytes, resulting in pigmentary incontinence [3, 22, 23]. These melanophages contain melanin aggregates that may be effectively targeted by picosecond lasers. In patients with stable disease, where active inflammation has subsided, the 1064‐nm wavelength used in our study provides deeper dermal penetration, allowing selective targeting of pigment clusters within the dermis while sparing the epidermis [15, 24].

A previous study examined Q‐switched Nd:YAG laser treatment in nine patients with LPP. After 6 sessions of laser administered at 2‐week intervals to representative 5 × 5 cm [2] lesions on covered sites, including the arms and forearms (three patients), shoulders (two patients), and abdomen (one patient). The treatment employed a toning protocol using low‐fluence parameters (fluence 3 J/cm [2], spot size 6 mm, frequency 10 Hz). The results demonstrated no statistically significant improvement in melanin index [9], which aligns with our findings.

However, a case report described a patient with facial LPP that responded to combination therapy including picosecond laser. Initial treatment with topical tacrolimus 0.1% and oral hydroxychloroquine 200 mg twice daily for 6 months stabilized the disease. Subsequently, 3 sessions of 1064‐nm Nd:YAG picosecond laser were performed at 4‐month intervals using both toning and fractional modes, leading to a marked reduction in hyperpigmentation and improvement in PGA score [25]. Another case report described a patient with stable facial LPP who underwent 10 sessions of 1064‐nm picosecond laser at approximately 1‐month intervals, followed by four additional 785‐nm picosecond laser sessions, resulting in marked pigment improvement without dyspigmentation or scarring [15]. Our study also showed an early significant reduction in melanin index at 2 months on the forehead, suggesting that picosecond laser may offer superior pigment clearance with optimized treatment parameters.

Therapeutic response to the picosecond laser may vary based on multiple factors. For example, all participants continued their previous topical and/or systemic treatments, which may have contributed to pigmentation improvement independently of laser treatment [7, 26, 27]. Therefore, the independent therapeutic effect of picosecond laser could not be fully isolated in this study. Despite the modest improvement, we found that participant‐reported satisfaction scores were significantly higher on the treated side compared to the control side (*p* < 0.05). Although this finding could reflect subtle aesthetic improvements (e.g., skin texture, rejuvenation) as well as the psychological confidence from receiving cosmetic treatment, combined with the absence of major adverse events, it should be interpreted cautiously because satisfaction was a secondary outcome assessed in an unblinded setting.

Hypopigmentation is a major concern when it comes to laser treatment in LPP. Confetti‐like leukoderma [10], scarring [10], and hypopigmentation [9] have been reported in previous studies using Q‐switched Nd:YAG lasers for LPP. In contrast, our study employed a picosecond laser toning protocol demonstrated an excellent safety profile, with only mild and transient side effects. No major adverse events occurred. Seven patients (63.6%) experienced a burning sensation, and three patients (27.3%) reported pruritus. The mean ± SD pain score was 3.7 ± 1.7. Our findings support that laser toning, characterized by low fluence, multi‐pass application, and a large spot size, minimizes the risk of epidermal disruption and cellular damage [28, 29]. Additionally, the 1064‐nm picosecond laser is safer for darker skin types, as its longer wavelength allows deeper dermal penetration while protecting epidermal pigmentation, thereby reducing the risk of post‐inflammatory hyperpigmentation (PIH) and erythema compared to nanosecond lasers [15, 30]. Taken together, these findings highlight the clinical utility of picosecond laser toning as a safe and well‐tolerated treatment option for LPP, particularly relevant given its higher prevalence among patients with darker skin types.

To our knowledge, this study is the first randomized controlled trial to examine the efficacy and safety of picosecond laser in LPP. However, there are some limitations. First, the small sample size may have limited the power to detect subtle pigmentation changes between the treated and control sides. Second, all participants continued background topical and/or systemic treatments during the study period, which prevented estimation of the independent effect of the picosecond laser. Due to the limited sample size, additional subgroup or sensitivity analyses stratified by background treatment type were not feasible. Third, the absence of a sham‐controlled arm prevented blinding in the assessment of patient satisfaction, which should therefore be interpreted with caution. Nonetheless, our finding of an early significant reduction in melanin index at 2 months on the forehead highlights its potential efficacy and supports further investigation. Further studies with larger sample sizes, sham‐controlled designs, stricter control of concomitant therapies, more treatment sessions, and optimized parameters (e.g., fluence, treatment intervals) are needed to confirm these findings and establish the role of picosecond laser as a promising therapeutic option for LPP.

## 5 Conclusion

While the 1064‐nm picosecond laser showed limited effectiveness in improving pigmentation in this study, it demonstrated a good safety profile. Larger studies with a sham‐controlled arm and strict control of concomitant therapies are needed to determine the independent therapeutic value of picosecond laser for LPP.

## Author Contributions

S.R., T.Y. designed the study. T.Y., N.S., S.S., and N.W. carried out the treatments and measurements. S.R., and K.T. contributed to the evaluation and interpretation of the results. S.S, T.Y. and S.R. wrote the manuscript. S.R. and K.T. supervised the project.

## Funding

This work was supported by The division of Dermatology, Faculty of Medicine Ramathibodi hospital, Mahidol university.

## Disclosure

Study Design: This study was a prospective, split‐face, randomized, controlled, evaluator‐blind study conducted at a university‐based hospital (Ramathibodi Hospital, Bangkok, Thailand) from November 2022 to February 2025. The study was approved by the Mahidol University Institutional Review Board for Ethics in Human Research (MURA2022/644). It was registered in the Thai Clinical Trials Registry (TCTR20250311004). Written informed consent was obtained from all participants before enrollment.

## Consent

The patient provided written informed consent for the case details and accompanying images to be published in this manuscript.

## Conflicts of Interest

The authors declare no conflicts of interest.

## Supporting information


**Table S1:** Mean modified dermal pigmentation area and severity index of picosecond laser treated sides and control sides assessed by a blinded dermatologist at each visit.
**Table S2:** Mean melanin index at forehead of picosecond laser treated sides and control sides.
**Table S3:** Mean melanin index at cheek of picosecond laser treated sides and control sides.
**Table S4:** Mean melanin index at neck of picosecond laser treated sides and control sides.

## Data Availability

The data that support the findings of this study are available on request from the corresponding author. The data are not publicly available due to privacy or ethical restrictions.

## References

[1] R. F. Wang , D. Ko , B. J. Friedman , H. W. Lim , and T. F. Mohammad , “Disorders of Hyperpigmentation. Part I. Pathogenesis and Clinical Features of Common Pigmentary Disorders,” Journal of the American Academy of Dermatology 88 (2023): 271–288. 35151757 10.1016/j.jaad.2022.01.051

[2] S. P. W. Kumarasinghe , A. Pandya , V. Chandran , et al., “A Global Consensus Statement on Ashy Dermatosis, Erythema Dyschromicum Perstans, Lichen Planus Pigmentosus, Idiopathic Eruptive Macular Pigmentation, and Riehls Melanosis,” International Journal of Dermatology 58 (2019): 263–272. 30176055 10.1111/ijd.14189

[3] S. Rutnin , S. Udompanich , N. Pratumchart , S. Harnchoowong , and V. Vachiramon , “Ashy Dermatosis and Lichen Planus Pigmentosus: The Histopathological Differences,” BioMed Research International 2019 (2019): 5829185. 31781623 10.1155/2019/5829185 PMC6855079

[4] A. Yadav , T. Garg , A. K. Mandal , and R. Chander , “Quality of Life in Patients With Acquired Pigmentation: An Observational Study,” Journal of Cosmetic Dermatology 17 (2018): 1293–1294. 29998547 10.1111/jocd.12686

[5] V. Gupta , D. Yadav , S. Satapathy , et al., “Psychosocial Burden of Lichen Planus Pigmentosus Is Similar to Vitiligo, but Greater Than Melasma: A Cross‐Sectional Study From a Tertiary‐Care Center in North India,” Indian Journal of Dermatology, Venereology and Leprology 87 (2021): 341–347. 33943064 10.25259/IJDVL_877_19

[6] N. Syder , K. Sicco , and D. Gutierrez , “Updates in Therapeutics for Lichen Planus Pigmentosus,” Journal of Drugs in Dermatology 21 (2022): 324–330. 35254753 10.36849/JDD.6454

[7] N. Al‐Mutairi and M. El‐Khalawany , “Clinicopathological Characteristics of Lichen Planus Pigmentosus and Its Response to Tacrolimus Ointment: An Open Label, Non‐Randomized, Prospective Study,” Journal of the European Academy of Dermatology and Venereology 24 (2010): 535–540. 19840200 10.1111/j.1468-3083.2009.03460.x

[8] S. K. Muthu , T. Narang , U. N. Saikia , A. J. Kanwar , D. Parsad , and S. Dogra , “Low‐Dose Oral Isotretinoin Therapy in Lichen Planus Pigmentosus: An Open‐Label Non‐Randomized Prospective Pilot Study,” International Journal of Dermatology 55 (2016): 1048–1054. 27062273 10.1111/ijd.13293

[9] N. Bhari , V. K. Sharma , S. Singh , A. Parihar , and S. Arava , “Effect of Q‐Switched Nd‐YAG Laser on the Clinical, Pigmentary, and Immunological Markers in Patients With Lichen Planus Pigmentosus: A Pilot Study,” Dermatology and Therapy 33 (2020): e13208. 10.1111/dth.13208 31885158

[10] D. S. D. Shah , D. S. Aurangabadkar , and D. B. Nikam , “An Open‐Label Non‐Randomized Prospective Pilot Study of the Efficacy of Q‐Switched Nd‐YAG Laser in Management of Facial Lichen Planus Pigmentosus,” Journal of Cosmetic and Laser Therapy 21 (2019): 108–115. 29768073 10.1080/14764172.2018.1469770

[11] R. Saluja and R. D. Gentile , “Picosecond Laser: Tattoos and Skin Rejuvenation,” Facial Plastic Surgery Clinics of North America 28 (2020): 87–100. 31779945 10.1016/j.fsc.2019.09.008

[12] B. Zysset , J. G. Fujimoto , C. A. Puliafito , R. Birngruber , and T. F. Deutsch , “Picosecond Optical Breakdown: Tissue Effects and Reduction of Collateral Damage,” Lasers in Surgery and Medicine 9 (1989): 193–204. 2659910 10.1002/lsm.1900090302

[13] C. S. M. Wong , M. W. M. Chan , S. Y. N. Shek , C. K. Yeung , and H. H. L. Chan , “Fractional 1064 Nm Picosecond Laser in Treatment of Melasma and Skin Rejuvenation in Asians, A Prospective Study,” Lasers in Surgery and Medicine 53 (2021): 1032–1042. 33544930 10.1002/lsm.23382

[14] T. Chalermchai and P. Rummaneethorn , “Effects of a Fractional Picosecond 1,064 Nm Laser for the Treatment of Dermal and Mixed Type Melasma,” Journal of Cosmetic and Laser Therapy 20 (2018): 134–139. 29020467 10.1080/14764172.2017.1376098

[15] A. Belzer , M. A. Swallow , M. Gowen , and K. C. Suozzi , “Picosecond Neodymium‐Doped Yttrium‐Aluminum‐Garnet Laser Therapy for Pigmentation due to Lichen Planus Pigmentosus in a Patient With Skin of Color,” JAAD Case Reports 34 (2023): 45–47. 36936863 10.1016/j.jdcr.2023.01.029 PMC10018222

[16] K. Vinay , G. Dabas , D. Parsad , and M. S. Kumaran , “A Novel Scale for Measurement of Acquired Dermal Macular Hyperpigmentation Severity,” Journal of the European Academy of Dermatology and Venereology 32 (2018): e251‐e3. 29283460 10.1111/jdv.14772

[17] M. S. Kumaran , G. Dabas , K. Vinay , and D. Parsad , “Reliability Assessment and Validation of the Dermal Pigmentation Area and Severity Index: A New Scoring Method for Acquired Dermal Macular Hyperpigmentation,” Journal of the European Academy of Dermatology and Venereology 33 (2019): 1386–1392. 30801771 10.1111/jdv.15516

[18] H. Alabdulrazzaq , J. A. Brauer , Y. S. Bae , and R. G. Geronemus , “Clearance of Yellow Tattoo Ink With a Novel 532‐Nm Picosecond Laser,” Lasers in Surgery and Medicine 47 (2015): 285–288. 25899971 10.1002/lsm.22354

[19] E. L. Tanzi , J. R. Lupton , and T. S. Alster , “Lasers in Dermatology: Four Decades of Progress,” Journal of the American Academy of Dermatology 49 (2003): 1–31. 12833005 10.1067/mjd.2003.582

[20] Y. Ge , Y. Yang , L. Guo , et al., “Comparison of a Picosecond Alexandrite Laser Versus a Q‐Switched Alexandrite Laser for the Treatment of Nevus of Ota: A Randomized, Split‐Lesion, Controlled Trial,” Journal of the American Academy of Dermatology 83 (2020): 397–403. 30885760 10.1016/j.jaad.2019.03.016

[21] W. Yu , J. Zhu , W. Yu , D. Lyu , X. Lin , and Z. Zhang , “A Split‐Face, Single‐Blinded, Randomized Controlled Comparison of Alexandrite 755‐Nm Picosecond Laser Versus Alexandrite 755‐Nm Nanosecond Laser in the Treatment of Acquired Bilateral Nevus of Ota‐Like Macules,” Journal of the American Academy of Dermatology 79 (2018): 479–486. 29288102 10.1016/j.jaad.2017.12.053

[22] E. Rieder , J. Kaplan , H. Kamino , M. Sanchez , and M. K. Pomeranz , “Lichen Planus Pigmentosus,” Dermatology Online Journal 19 (2013): 20713. 24365004

[23] A. A. Gru and A. L. Salavaggione , “Lichenoid and Interface Dermatoses,” Seminars in Diagnostic Pathology 34 (2017): 237–249. 28396069 10.1053/j.semdp.2017.03.001

[24] T. Ohshiro , T. Ohshiro , K. Sasaki , and K. Kishi , “Picosecond Pulse Duration Laser Treatment for Dermal Melanocytosis in Asians: A Retrospective Review,” Laser Therapy 25 (2016): 99–104. 27721561 10.5978/islsm.16-OR-07 PMC4961675

[25] C. Y. Wu and F. L. Lin , “A Successful Combination Therapy of Tacrolimus, Hydroxychloroquine and Picosecond Laser for Lichen Planus Pigmentosus,” Australasian Journal of Dermatology 60 (2019): e336‐e7. 31012083 10.1111/ajd.13060

[26] H. M. Cheng , S. Y. Chuah , E. Y. Gan , A. Jhingan , and S. T. G. Thng , “A Retrospective Clinico‐Pathological Study Comparing Lichen Planus Pigmentosus With Ashy Dermatosis,” Australasian Journal of Dermatology 59 (2018): 322–327. 29635779 10.1111/ajd.12813

[27] Y. Chen , L. Xue , D.‐j. Guo , et al., “Combination Therapy of Acitretin Capsule and Chinese Herbs for Patients With Lichen Planus Pigmentosus‐Inversus,” Chinese Journal of Integrative Medicine 25 (2019): 922–925. 31444667 10.1007/s11655-019-3033-7

[28] T. Omi , R. Yamashita , S. Kawana , S. Sato , and Z. Naito , “Low Fluence Q‐Switched Nd: YAG Laser Toning and Q‐Switched Ruby Laser in the Treatment of Melasma: A Comparative Split‐Face Ultrastructural Study,” Laser Therapy 21 (2012): 15–24. 10.5978/islsm.12-OR-03 PMC3944591 24610976

[29] J. K. Hong , S. H. Shin , S. J. Park , S. J. Seo , and K. Y. Park , “A Prospective, Split‐Face Study Comparing 1,064‐Nm Picosecond Nd:YAG Laser Toning With 1,064‐Nm Q‐Switched Nd:YAG Laser Toning in the Treatment of Melasma,” Journal of Dermatological Treatment 33 (2022): 2547–2553. 35067157 10.1080/09546634.2022.2033674

[30] J. Feng and L. Huang , “Comparison of Picosecond and Nanosecond Nd:YAG 1064‐Nm Lasers in the Treatment of Melasma: A Split‐Face Randomized Clinical Trial,” Plastic and Reconstructive Surgery 151 (2023): 772–777. 36729879 10.1097/PRS.0000000000009994

