# Supplementary material for: Efficacy of 1064‐nm Picosecond Laser in the Treatment of Lichen Planus Pigmentosus: A Split‐Face Randomized Controlled Trial
Source: J Cosmet Dermatol. 2026 Apr 7;25(4):e70846. doi: 10.1111/jocd.70846 (PMC13058402; doi:10.1111/jocd.70846)
Supplement: Supplementary file 1 — Table S1: Mean modified dermal pigmentation area and severity index of picosecond laser treated sides and control sides assessed by a blinded dermatologist at each visit. Table S2: Mean melanin index at forehead of picosecond laser treated sides and control sides. Table S3: Mean melanin index at cheek of picosecond laser treated sides and control sides. Table S4: Mean melanin index at neck of picosecond laser treated sides and control sides. [file JOCD-25-e70846-s001.docx]

**Supplementary Table 1** Mean modified dermal pigmentation area and severity index of picosecond laser treated sides and control sides assessed by a blinded dermatologist at each visit

| **Visit** | **Month** | **Picosecond laser (mean ± SD)** | **Control (mean ± SD)** | **p-value** |
| --- | --- | --- | --- | --- |
| 1 (Baseline) | 0 | 7.17 ± 4.04 | 7.19 ± 4.02 | 0.883 |
| 2 | 1 | 6.91 ± 4.13 | 7.13 ± 4.08 | 0.479 |
| 3 | 2 | 6.72 ± 3.89 | 7.07 ± 4.13 | 0.267 |
| 4 | 3 | 6.26 ± 3.97 | 6.75 ± 3.99 | 0.131 |
| 5 | 4 | 5.66 ± 3.72 | 6.05 ± 4.06 | 0.229 |
| 6 | 6 | 5.40 ± 3.79 | 5.87 ± 3.79 | 0.149 |
| 7 | 9 | 5.15 ± 3.42 | 5.52 ± 3.45 | 0.245 |

**Supplementary Table 2** Mean melanin index at forehead of picosecond laser treated sides and control sides

| **Visit** | **Month** | **Picosecond laser (mean ± SD)** | **Control (mean ± SD)** | **p-value** |
| --- | --- | --- | --- | --- |
| 1 (Baseline) | 0 | 0.81 ± 0.14 | 0.83 ± 0.15 | 0.092 |
| 2 | 1 | 0.77 ± 0.14 | 0.80 ± 0.15 | 0.060 |
| 3 | 2 | 0.78 ± 0.14 | 0.82 ± 0.15 | 0.011* |
| 4 | 3 | 0.79 ± 0.15 | 0.80 ± 0.14 | 0.478 |
| 5 | 4 | 0.79 ± 0.15 | 0.80 ± 0.15 | 0.505 |
| 6 | 6 | 0.80 ± 0.15 | 0.82 ± 0.17 | 0.187 |
| 7 | 9 | 0.82 ± 0.16 | 0.82 ± 0.15 | 0.655 |

**Supplementary Table 3** Mean melanin index at cheek of picosecond laser treated sides and control sides

| **Visit** | **Month** | **Picosecond laser (mean ± SD)** | **Control (mean ± SD)** | **p-value** |
| --- | --- | --- | --- | --- |
| 1 (Baseline) | 0 | 0.81 ± 0.12 | 0.80 ± 0.13 | 0.789 |
| 2 | 1 | 0.80 ± 0.13 | 0.80 ± 0.14 | 0.582 |
| 3 | 2 | 0.81 ± 0.13 | 0.81 ± 0.14 | 0.818 |
| 4 | 3 | 0.80 ± 0.13 | 0.80 ± 0.15 | 0.574 |
| 5 | 4 | 0.81 ± 0.14 | 0.80 ± 0.15 | 0.608 |
| 6 | 6 | 0.81 ± 0.16 | 0.81 ± 0.15 | 0.693 |
| 7 | 9 | 0.83 ± 0.16 | 0.83 ± 0.17 | 0.766 |

**Supplementary Table 4** Mean melanin index at neck of picosecond laser treated sides and control sides

| **Visit** | **Month** | **Picosecond laser (mean ± SD)** | **Control (mean ± SD)** | **p-value** |
| --- | --- | --- | --- | --- |
| 1 (Baseline) | 0 | 0.75 ± 0.12 | 0.74 ± 0.13 | 0.409 |
| 2 | 1 | 0.78 ± 0.12 | 0.78 ± 0.13 | 0.926 |
| 3 | 2 | 0.78 ± 0.12 | 0.77 ± 0.11 | 0.788 |
| 4 | 3 | 0.76 ± 0.12 | 0.77 ± 0.13 | 0.704 |
| 5 | 4 | 0.76 ± 0.11 | 0.77 ± 0.11 | 0.571 |
| 6 | 6 | 0.77 ± 0.12 | 0.78 ± 0.13 | 0.609 |
| 7 | 9 | 0.78 ± 0.12 | 0.78 ± 0.13 | 0.891 |
